# Glycosylated Receptor-Binding-Domain-Targeting Mucosal Vaccines Protect Against SARS-CoV-2 Omicron and MERS-CoV

**DOI:** 10.3390/vaccines13030293

**Published:** 2025-03-10

**Authors:** Xiaoqing Guan, Abhishek K. Verma, Qian Liu, Melissa Palacios, Abby E. Odle, Stanley Perlman, Lanying Du

**Affiliations:** 1 Institute for Biomedical Sciences, Georgia State University, Atlanta, GA 30303, USA; 2 Department of Microbiology and Immunology, University of Iowa, Iowa City, IA 52242, USA; 3 Department of Pediatrics, University of Iowa, Iowa City, IA 52242, USA

**Keywords:** pathogenic coronavirus, COVID-19, SARS-CoV-2, MERS-CoV, receptor-binding domain, mucosal immunity, protective efficacy

## Abstract

Background. The pathogenic coronaviruses (CoVs) MERS-CoV and SARS-CoV-2, which are responsible for the MERS outbreak and the COVID-19 pandemic, respectively, continue to infect humans, with significant adverse outcomes. There is a continuing need to develop mucosal vaccines against these respiratory viral pathogens to prevent entry and replication at mucosal sites. The receptor-binding domain (RBD) of the CoV spike (S) protein is a critical vaccine target, and glycan masking is a unique approach for designing subunit vaccines with improved neutralizing activity. Methods. We evaluated the efficacy of mucosal immunity, broad neutralizing activity, and cross-protection afforded by a combined glycosylated mucosal subunit vaccine encoding the RBDs of the original SARS-CoV-2 strain (SARS2-WT-RBD), the Omicron-XBB.1.5 variant (SARS2-Omi-RBD), and MERS-CoV (MERS-RBD). Results. Intranasal administration of the three-RBD protein cocktail induced effective, durable IgA and systemic IgG antibodies specific for the S protein of these CoVs, thereby neutralizing infection by pseudotyped SARS-CoV-2-WT, Omicron-XBB.1.5, and MERS-CoV. The mucosal vaccine cocktail protected immunized mice from challenge with SARS-CoV-2 Omicron-XBB.1.5 and MERS-CoV, leading to a significant reduction in the viral titers in the lungs. By contrast, the individual glycosylated RBD proteins only induced such immune responses and neutralizing antibodies against either SARS-CoV-2 or MERS-CoV, protecting against subsequent challenge with either SARS-CoV-2 or MERS-CoV; they did not provide simultaneous protection against both CoVs. Conclusions. This study describes a unique strategy for designing efficacious mucosal subunit vaccines that induce durable mucosal immunity, cross-neutralizing activity, and cross-protection against SARS-CoV-2 and MERS-CoV, highlighting the potential for the design of mucosal vaccines against other pathogens.

## 1. Introduction

Severe acute respiratory syndrome coronavirus-2 (SARS-CoV-2), a highly contagious pathogenic human CoV first reported in late 2019, caused the global pandemic of CoV Disease 2019 (COVID-19) that lasted for several years [1,2,3]. SARS-CoV-2 is transmitted easily among humans; indeed, a report by the World Health Organization shows that, as of 2 February 2025, at least 777.36 million people have been infected, with more than 7.08 million deaths [4,5,6]. This original SARS-CoV-2 strain mutated rapidly (and continues to do so), leading to emergence of a number of variants of interest and five major variants of concern, including the Alpha, Beta, Gamma, Delta, and Omicron variants (as well as many subvariants of Omicron) [7,8,9,10,11].

Middle East respiratory syndrome CoV (MERS-CoV), first identified in April, 2012, is another highly pathogenic human CoV that causes Middle East respiratory syndrome (MERS) [12]. MERS-CoV is a zoonotic virus that uses dromedary camels as an intermediate reservoir [13,14]. People become infected via direct transmission from camels [15,16,17]. Unlike SARS-CoV-2, MERS-CoV human-to-human transmissibility requires close contact; thus, it has caused sporadic outbreaks in health care workers or in community settings [18,19,20,21]. Although MERS-CoV is less transmissible between humans than SARS-CoV-2, the mortality rate is much higher (>36%) than that of SARS-CoV-2 (~9%). As of 5 February 2025, 2626 MERS cases, including 953 deaths, have been reported globally [22].

The surface spike (S) proteins of SARS-CoV-2 and MERS-CoV (both of which belong to the Beta genus of CoVs) play essential roles in initial viral entry and subsequent infection of host cells [23,24,25]. The native S protein forms a trimer, and each trimer comprises two subunits: S1, which includes the receptor-binding domain (RBD), and S2 [25,26,27,28,29]. The RBDs of SARS-CoV-2 and MERS-CoV mediate viral entry by binding to a specific cellular receptor (angiotensin-converting enzyme 2 (ACE2) for SARS-CoV-2 and dipeptidyl peptidase 4 (DPP4) for MERS-CoV), and the S2 subunit then facilitates membrane fusion [25,29,30,31,32]. Therefore, the S proteins (particularly the RBDs) of these CoVs are a key target for vaccine development. Because SARS-CoV-2 and MERS-CoV are a continual threat to public health worldwide, there is a need to develop effective countermeasures, including vaccines, with broad-spectrum efficacy against both viral infections.

SARS-CoV-2 and MERS-CoV are two important mucosal viral pathogens that initiate infection at mucosal sites in the upper respiratory tract. Thus, it is critical to develop vaccines capable of inducing effective mucosal immune responses to inhibit/prevent the initial stages of infection. The glycan masking technique is a unique strategy used for epitope-focused vaccine design. The technique involves adding an N-linked glycan probe to the surface of an antigen to mask regions of non-neutralizing epitopes (i.e., the epitopes without neutralizing potential), thereby focusing the immune response on highly neutralizing epitopes [33]. Fusion of an Fc tag of human IgG to protein vaccines may help proteins interact with the Fc receptor on antigen presenting cells, thereby improving their overall immunogenicity and mucosal immunity [34].

Here, we designed a glycan-masked subunit vaccine encoding the Fc-fused RBD of SARS-CoV-2 Omicron variant (XBB.1.5) and explored its ability to induce durable, mucosal immune responses. We also examined its broadly protective efficacy when administered together (as a cocktail) with glycan-masked Fc-fused proteins targeting the RBDs of the original SARS-CoV-2 strain and MERS-CoV [35,36].

## 2. Materials and Methods

### 2.1. Cell Culture

Vero, Vero E81, HEK293T cells, Huh-7 cells (ATCC, Manassas, VA, USA), and HEK293T cells expressing human ACE2 (hACE2-293T, Laboratory stock) supplied with Dulbecco’s Modified Eagle Medium (DMEM) cell culture medium, 10% fetal bovine serum (FBS) (R&D Systems, Minneapolis, MN, USA), and 1% Penicillin-Streptomycin (P/S, Thermo Fisher Scientific, Waltham, MA, USA) were cultured at 37 °C with 5% CO_2_. HEK293F cells (Thermo Fisher Scientific) supplied with serum-free medium (ESF SFM) (Expression Systems, Davis, CA, USA) were cultured in a 37 °C Orbital Shaker incubator with 8% CO_2_ and 120 rpm rotation.

### 2.2. Recombinant Constructs and Protein Preparation

Recombinant DNA encoding the RBD of the SARS-CoV-2 Omicron variant (XBB.1.5 subvariant) with an N-linked glycosylation (at residues 515/517) (i.e., SARS2-Omi-RBD) was amplified via polymerase chain reaction (PCR) based on plasmid encoding codon-optimized sequences of the SARS-CoV-2 S protein (GenBank Accession No. WCZ72555.1). The purified PCR product was fused into a C-terminal Fc tag of human IgG in a vector. A Fc-fused RBD of the original SARS-CoV-2 strain (GenBank Accession No. QHR63250.2) with an N-linked glycosylation (at residues 519/521) (i.e., SARS2-WT-RBD) and an Fc-fused RBD of MERS-CoV (GenBank Accession No. AFS88936.1) with an N-linked glycosylation (at residue 579) (i.e., MERS-RBD) were constructed as previously described [35,36]. The recombinant plasmids with confirmed sequences were transfected into HEK293F cells in the presence of linear Polyethylenimine Hydrochloride (PEI) transfection reagent (MW 40,000, PEI MAX) (Polysciences, Warrington, PA, USA: 24765). The mass ratio of PEI to plasmid was 3:1. Four to five days after transfection, cell culture supernatant containing the expressed proteins was collected via centrifugation for 20 min at 6000× *g*. The expressed proteins were purified using nProtein A Sepharose 4 Fast Flow (Cytiva, Marborough, MA, USA), which was followed by concentration using an Amicon Ultra Centrifugal Filter (10 kDa MWCO; MilliporeSigma, Burlington, MA, USA: UFC9010) in the presence of PBS (pH 7.4, containing 137 mM NaCl, 2.7 mM KCl, 8 mM Na_2_HPO_4_, and 1.47 mM KH_2_PO_4_).

### 2.3. Protein Stability

The stability of above purified proteins was measured via thermal shift assay in buffer solutions at variable pH values in the presence of sodium chloride (NaCl, 200 mM) via the CFX Opus 96 Real-Time PCR System according to the manufacturer’s protocol (Bio-Rad, Hercules, CA, USA). Briefly, individual proteins (5 µg/10 µL/protein) were loaded into each well of a 96-well PCR plate, followed by addition of SYPRO Orange Protein Gel Stain (2.5 µL/well) (MilliporeSigma). Tris buffer (12.5 µL/well) at different pH values (5 to 9) and NaCl (200 mM) were then loaded into each well. The melting temperature of each protein in the respective buffer was measured using the above Real-Time PCR System.

### 2.4. Mouse Immunization and Sample Collection

Groups of five female wildtype BALB/c mice (at 6–8 weeks of age) were used for immunization experiments. The mice were purchased from the Jackson Laboratory and randomly assigned to the following vaccination groups. To evaluate the vaccine’s immune responses, mice were intranasally (i.n.) immunized with each individual protein (10 μg/mouse), including SARS2-WT-RBD, SARS2-Omi-RBD, and MERS-RBD, their combination (i.e., a cocktail containing 3.33 μg of each protein), or PBS control, in the presence of Poly(I:C) adjuvant (10 μg/mouse; InvivoGen, San Diego, CA, USA). The total volume of the vaccine for each mouse was 20 µL (10 µL for each nostril). After being anesthetized, the mouse was held by the scruff and ears, and the dose was applied with a micropipette into the nostrils drop by drop to allow the mouse to inhale the inoculum. The mice were boosted for two doses at a 3-week interval. A total of 32 weeks after the third immunization, the mice were further boosted with the same immunogens described above. Serum and bronchoalveolar lavage (BAL) samples were collected 14 days after the last immunization for analysis of specific antibodies via enzyme-linked immunosorbent assay (ELISA) and/or neutralizing antibodies via pseudovirus neutralization assay, as described below.

### 2.5. ELISA

ELISA was performed to test specific IgG and subtype (IgG1 and IgG2a) antibodies in immunized mouse sera, as well as IgA antibodies in collected BAL fluid. Briefly, His-tagged S proteins of the original SARS-CoV-2 strain, Omicron-XBB.1.5 variant, or MERS-CoV (1 μg/mL) were coated to ELISA plates, which were stored overnight at 4 °C. After the addition of blocking buffer (i.e., 2% fat-free milk in PBS containing 0.1% Tween-20 (PBST)) to block non-specific binding, the plates were incubated for 1 h at 37 °C, followed by a single wash with PBST. The plates were then incubated with serially diluted mouse sera or BAL fluid for 2 h at 37 °C. After 4 washes with PBST, the plates were incubated with horseradish peroxidase (HRP)-conjugated anti-mouse IgG (1:25,000), anti-mouse-IgG1 (1:15,000), anti-mouse-IgG2a (1:2000), or anti-mouse IgA (1:3000) antibody (Thermo Fisher Scientific) for 1 h at 37 °C. After 4 washes with PBST, the plates were incubated with TMB (3,3′,5,5′-Tetramethylbenzidine) substrate (MilliporeSigma), followed by termination of the reaction with sulfuric acid (1 N). The plates were measured at absorbance 450 nm (OD_450_) using a Cytation 7 microplate reader (BioTek Instruments, Winooski, VT, USA). The OD_450_ values were analyzed with nonlinear regression, and the titer of each antibody was interpolated (the cutoff: 4 times of the blank).

### 2.6. Pseudovirus Generation and Neutralization Assay

Pseudoviruses, including SARS-CoV-2 (original strain and Omicron-XBB.1.5 variant) and MERS-CoV, were generated from HEK293T cells co-transfected with pLenti-CMV-LUC and psPAX2 (Addgene, Watertown, MA, USA) plasmids and a recombinant plasmid encoding the respective S proteins of SARS-CoV-2 or MERS-CoV using PEI transfection reagent. A total of 6–8 h after transfection, the medium was replaced with fresh culture medium containing 10% FBS. A total of 72 hours after transfection, the pseudovirus-containing cell culture supernatant was harvested by centrifugation for 5 min at 2000 rpm. The pseudovirus neutralization assay was performed via the incubation of serially diluted mouse sera with the respective pseudoviruses collected above for 1 h at 37 °C, followed by addition to hACE2-293T (for SARS-CoV-2 pseudovirus) or Huh-7 (for MERS-CoV pseudovirus) cells, which were pre-seeded in 96-well cell culture plates. Fresh medium was added to the cells 24 h later, and the cells were cultured for an additional 48 h, followed by lysis with Cell Lysis buffer (Promega, Madison, MI, USA). The cell lysates were transferred to white microplates, followed by the addition of luciferase substrate (Promega) and the measurement of relative luciferase activity using the Cytation 7 microplate reader. The serum neutralization titer (NT_50_) was calculated as the serum dilution at which pseudovirus was neutralized by 50%.

### 2.7. Virus Challenge and Evaluation Studies

Immunized mice were challenged intranasally (i.n.) with SARS-CoV-2 or MERS-CoV 10 days after the 2nd boost immunization and evaluated for protective efficacy as described below. For the SARS-CoV-2 challenge, mice were infected with the Omicron variant (XBB.1.5; 10^5^ plaque-forming unit (PFU)/50 μL/mouse), and the lungs were collected from the infected mice 2 days later. For the MERS-CoV challenge, mice were first i.n. transduced with Ad5-hDPP4 (Ad5CMV/hDPP4-myc-flag vector; 2.5 × 10^8^ focus-forming unit) and then i.n. infected with MERS-CoV (EMC2012 strain; 10^5^ PFU/50 μL/mouse) 5 days later, followed by collection of lungs 3 days after infection. Since the SARS-CoV-2 Omicron variant and MERS-CoV were not lethal to wildtype mice (for SARS-CoV-2 challenge) or Ad5-hDPP4-transduced wildtype mice (for MERS-CoV challenge), viral titers were tested in the collected lung tissues via plaque assay. Specifically, serially diluted supernatant of homogenized lung tissues was incubated with Vero cells (with ACE2 and TMPRSS2; for SARS-CoV-2) or Vero E81 cells (for MERS-CoV), supplied with DMEM cell culture medium for 1 h at 37 °C. After removing the medium, the cells were overlaid with 0.6% agarose and cultured for three additional days. After removing the overlays from the cells, the plaques were visualized after staining with 0.1% crystal violet (Fisher Scientific, Hampton, NH, USA). Viral titers in the lungs of mice were reported as the PFU/mL of lung tissues.

### 2.8. Quantification and Statistical Analysis

Statistical calculations were performed using GraphPad Prism 9 software. Ordinary one-way ANOVA analysis was used to compare the statistical differences among various groups and Tukey’s multiple comparison test was further applied to compare the difference between groups. *, **, ***, and **** illustrate *p* < 0.05, *p* < 0.01, *p* < 0.001, and *p* < 0.0001, respectively.

## 3. Results

### 3.1. Characterization of the Glycosylated RBD Subunit Vaccines and Mucosal Immunization

**Figure 1:**
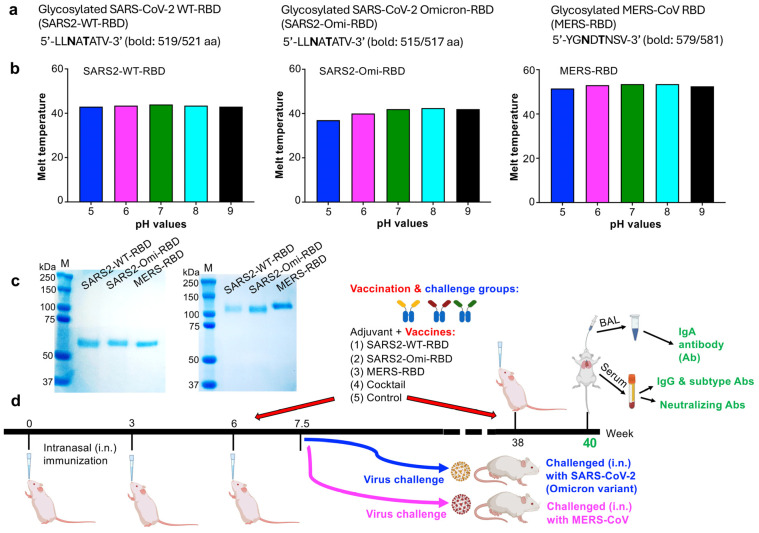
Protein characterization, immunization, and virus challenge schedules. (**a**) Summary of N-linked glycosylation sites (shown in bold) in the RBD regions. (**b**) Characterization of glycosylated RBD mucosal subunit vaccines via the thermal shift assay. The recombinant proteins, including SARS-CoV-2 WT-RBD (i.e., SARS2-WT-RBD), SARS-CoV-2-XBB.1.5-RBD (i.e., SARS2-Omi-RBD), and MERS-CoV RBD (i.e., MERS-RBD), were characterized for stability via the thermal shift assay at variable pH values in the presence of sodium chloride (200 mM NaCl). (**c**) SDS-PAGE images of glycosylated RBD proteins with (left) or without (right) boiling. Protein molecular weight markers (M) are shown on the left. (**d**) Mouse immunization and virus challenge schedules. BALB/c mice were intranasally (i.n.) immunized with the respective Fc-fused SARS2-WT-RBD, SARS2-Omi-RBD, and MERS-RBD proteins alone, their cocktail, or PBS control in the presence of Poly (I:C) mucosal adjuvant at 3-week intervals. Ten days after the 2nd boost (i.e., 7.5 weeks), the immunized mice were challenged (i.n.) with SARS-CoV-2 (Omicron-XBB.1.5 variant) or MERS-CoV (EMC2012 strain) and evaluated for protective efficacy via measurement of viral titers in the lungs. Additional mice were boosted at 38 weeks and then two weeks later, before being evaluated for IgA antibodies (Abs) in bronchoalveolar lavage (BAL) fluid, as well as total IgG Abs, IgG subtype Abs, and neutralizing Abs in the sera.

Fc-fused proteins containing the N-linked glycosylated RBD of the Omicron variant (XBB.1.5; SARS2-Omi-RBD), the N-linked glycosylated RBD of the original SARS-CoV-2 strain (SARS2-WT-RBD), and the N-linked glycosylated RBD of MERS-CoV (MERS-RBD) (Figure 1a) were expressed in HEK293F cells and purified from the culture supernatant using protein A beads. The thermal shift assay, a fluorescence-dye-based method for determining the protein melting point at which the protein is denatured, was used to assess the stability of the protein vaccines in the buffers at different pH values and salt concentrations. All three proteins were stable upon exposure to various pH values (5–9) in the presence of 200 mM NaCl, with the melting temperatures staying in similar ranges for SARS2-WT-RBD (43–44 °C), SARS2-Omi-RBD (37–42.5 °C), and MERS-RBD (51.5–53.5 °C), respectively (Figure 1b). So, they are applicable for subsequent vaccination. The sizes of these proteins are about 60–70 kDa and 120–130 kDa, respectively, for boiled (Figure 1c, left) and non-boiled (Figure 1c, right) samples.

BALB/c mice were immunized with each protein individually or with a cocktail containing all three proteins via the intranasal route. The mice were boosted three times, with a 3-week interval between the first two boosts and a 32-week interval between the second and third boosts. IgA and IgG antibody responses, as well as neutralizing antibody levels in the serum, were then assessed (Figure 1d). Some mice were also evaluated after receiving only the first two boosts (with a 3-week interval) to assess protective efficacy against infection of SARS-CoV-2 Omicron (XBB.1.5) variant and MERS-CoV (Figure 1d).

### 3.2. The Glycosylated RBD Mucosal Subunit Vaccines Elicited Durable Systemic and Mucosal Antibody Responses

To evaluate whether the glycosylated RBD mucosal vaccines, or a combination of these, induce durable systemic and mucosal immune responses, we collected serum and BAL samples from immunized mice at 40 weeks post-immunization and measured serum IgG (including IgG subtypes) responses and mucosal IgA antibody responses.

**Figure 2:**
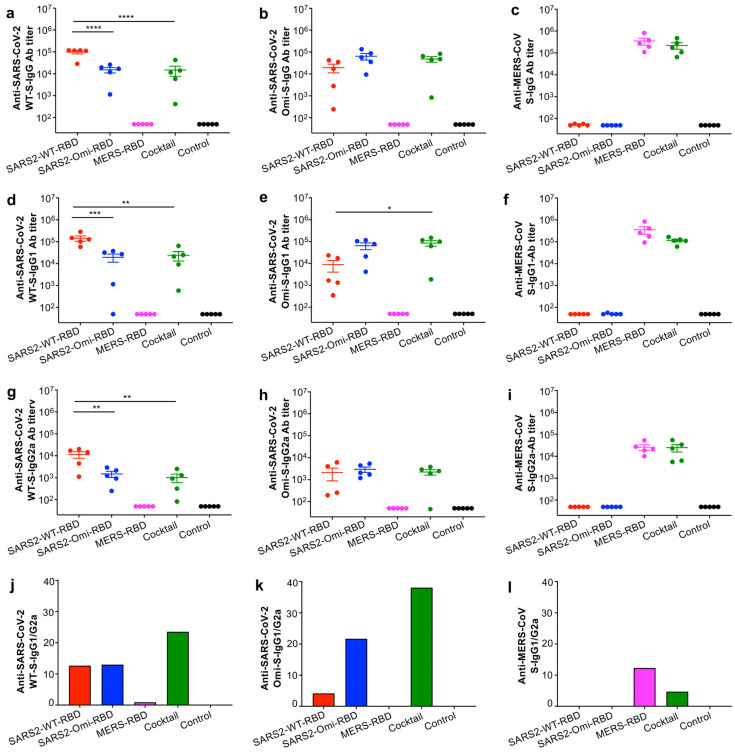
Serum IgG and subtype antibody responses induced by the glycosylated RBD mucosal subunit vaccines. BALB/c mice were immunized (i.n.) with each protein, their cocktail, or PBS control in the presence of the mucosal adjuvant, as described in Figure 1. The sera collected two weeks after the last dose were tested via ELISA for IgG (**a**–**c**), IgG1 (**d**–**f**), and IgG2a (**g**–**i**) antibodies (Abs) specific to the respective S proteins of the SARS-CoV-2 original strain (WT) (**a**,**d**,**g**), SARS-CoV-2 Omicron-XBB.1.5 (**b**,**e**,**h**), or MERS-CoV (**c**,**f**,**i**). The IgG and subtype Ab titers are expressed as mean ± standard deviation of the mean (s.e.m.) of five mice per group. The ratios of IgG1/IgG2a to the respective proteins are shown (**j**–**l**). Statistical significance among different vaccination groups is reported as * (*p* < 0.05), ** (*p* < 0.01), *** (*p* < 0.001), and **** (*p* < 0.0001), respectively. The experiments were repeated once with similar results.

The results showed that the glycosylated SARS2-WT-RBD or SARS2-Omi-RBD proteins (10 μg protein/mouse) alone induced effective IgG antibody responses against the respective S proteins of the original SARS-CoV-2 strain (WT) and the Omicron (XBB.1.5) variant, but they failed to induce strong IgG antibody responses against the S protein of MERS-CoV (Figure 2a–c). Similarly, the glycosylated MERS-RBD protein (10 μg protein/mouse) induced strong IgG antibody responses against the S protein of MERS-CoV but not against the S proteins of SARS-CoV-2 WT and Omicron (XBB.1.5) (Figure 2a–c). By contrast, vaccination with the cocktail (10 μg protein/mouse; 3.33 μg/RBD of the three RBDs) elicited potent IgG antibody responses against all three S proteins, with similar titers of IgG specific for the Omicron and MERS-CoV S proteins (Figure 2a–c). The reduced dosage of each protein within the cocktail (to 1/3 of that used for the individual vaccinations) meant that the titer of IgG antibodies specific for the S protein of SARS-CoV-2-WT induced by the cocktail was significantly lower than the titer of IgG antibodies specific for the S protein induced by the individual SARS2-WT-RBD protein (Figure 2a). In addition, the level of IgG1 or IgG2a antibodies induced by the cocktail comprising the three glycosylated RBD proteins, as well as by individual RBD proteins, tended to be similar to that of total IgG (Figure 2d–i). Overall, the immunization elicited higher IgG1 antibody titers than IgG2a antibody titers specific to the respective S proteins, leading to high IgG1/IgG2a ratios (Figure 2j–l).

**Figure 3:**
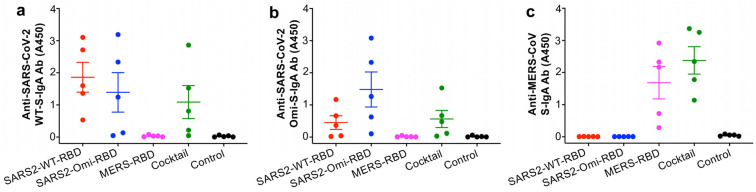
Mucosal IgA antibody responses induced by the glycosylated RBD mucosal subunit vaccines. BALB/c mice were immunized (i.n.) with each protein, their cocktail, or PBS control in the presence of the mucosal adjuvant, as described in Figure 1. The BAL fluid collected two weeks after the last dose were tested via ELISA for IgA antibodies (Abs) specific to the respective S proteins of the SARS-CoV-2 original strain (WT) (**a**), SARS-CoV-2 Omicron-XBB.1.5 variant (**b**), or MERS-CoV (**c**). The IgA Ab responses are shown as absorbance values at 450 nm (A450) and expressed as the mean ± s.e.m of five mice per group. The experiments were repeated once with similar results.

The glycosylated SARS2-WT-RBD and SARS2-Omi-RBD proteins induced effective mucosal IgA antibody responses against the SARS-CoV-2 WT and Omicron variant S proteins, but they did not induce IgA antibody responses against the MERS-CoV S protein (Figure 3a–c). In addition, the glycosylated MERS-RBD protein elicited MERS-CoV S-specific IgA antibody responses but did not induce IgA antibody responses against the SARS-CoV-2 WT and Omicron S proteins (Figure 3a–c). By contrast, the cocktail comprising all three RBD proteins elicited similar levels of IgA antibodies against all three S proteins, even though the dose of each protein in the cocktail was 1/3 that used for the individual proteins (Figure 3a–c).

The above data demonstrate that although the individual glycosylated RBD proteins induced effective and durable systemic IgG and mucosal IgA antibody responses against individual CoVs or cross-reacted with two strains belonging to the same CoV category (i.e., SARS2-WT-RBD and SARS2-Omi-RBD), the cocktail elicited potent and durable systemic and mucosal antibody responses against each of the original SARS-CoV-2 strain, the Omicron variant, and MERS-CoV.

### 3.3. The Glycosylated RBD Cocktail Subunit Mucosal Vaccine Elicits Durable and Broadly Neutralizing Antibody Responses Against SARS-CoV-2 and MERS-CoV

**Figure 4:**
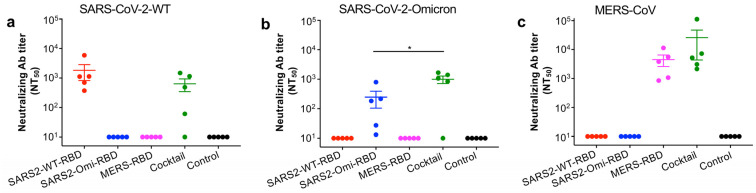
Serum neutralizing antibody responses induced by the glycosylated RBD mucosal subunit vaccines. BALB/c mice were immunized (i.n.) with each protein, their cocktail, or PBS control in the presence of the mucosal adjuvant, as described in Figure 1. Sera collected two weeks after the last dose were tested via pseudovirus neutralization assay for neutralizing antibodies (Abs) against pseudoviruses encoding the respective S proteins of SARS-CoV-2 original strain (WT) (**a**), SARS-CoV-2 Omicron-XBB.1.5 variant (**b**), or MERS-CoV (EMC2012 strain) (**c**). The 50% neutralizing Ab titers (NT_50_) are calculated based on the serum dilution at which pseudovirus was neutralized by 50% and expressed as mean ± s.e.m of five mice per group. Statistical significance among different vaccination groups is reported as * (*p* < 0.05). The experiments were repeated once with similar results.

To evaluate whether the glycosylated RBD mucosal vaccines (or a combination of all three) induce durable neutralizing antibody responses, we collected serum samples from mice at 40 weeks post-immunization and tested them for neutralizing activity against pseudotyped SARS-CoV-2 and MERS-CoV viruses. Notably, the SARS2-WT-RBD protein elicited neutralizing antibodies against the pseudotyped original SARS-CoV-2 strain but not against the pseudotyped SARS-CoV-2 Omicron-XBB1.5 or MERS-CoV viruses (Figure 4a–c). The SARS2-Omi-RBD protein elicited neutralizing antibodies against the pseudotyped SARS-CoV-2 Omicron-XBB1.5 variant but not against the pseudotyped original SARS-CoV-2 strain or MERS-CoV viruses (Figure 4a–c), and the MERS-RBD protein elicited neutralizing antibodies against the pseudotyped MERS-CoV but not against the pseudotyped original SARS-CoV-2 strain or Omicron-XBB1.5 variant (Figure 4a–c). By contrast, neutralizing antibodies induced by the cocktail of the three RBD proteins neutralized the pseudotyped original SARS-CoV-2 strain, Omicron-XBB.1.5 variant, and MERS-CoV (Figure 4a–c). These data indicate that while the individual glycosylated RBD proteins induced effective and durable neutralizing antibody responses against either SARS-CoV-2 (original strain and Omicron variant) or MERS-CoV, the cocktail of the three RBD proteins elicited efficient and durable neutralizing antibodies against all three viruses, suggesting broad-spectrum neutralizing activity against multiple pathogenic CoVs.

### 3.4. The Glycosylated RBD Cocktail Subunit Mucosal Vaccine Protects Mice from Challenge with SARS-CoV-2 Omicron and MERS-CoV

**Figure 5:**
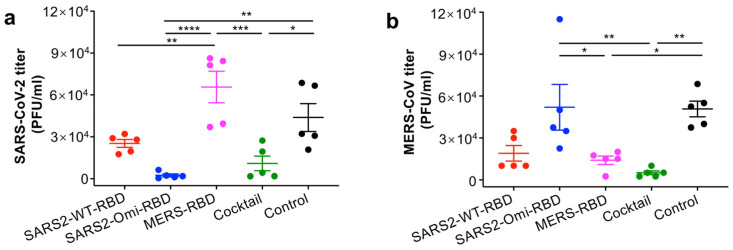
Protective efficacy induced by the glycosylated RBD mucosal subunit vaccines. BALB/c mice were immunized (i.n.) with each protein, their cocktail, or PBS control in the presence of the mucosal adjuvant, as described in Figure 1. At 10 days after the 2nd boost immunization, the mice were respectively challenged (i.n.) with SARS-CoV-2 (Omicron-XBB.1.5 variant) (**a**) or MERS-CoV (EMC2012 strain) (**b**), and their lungs were then collected for evaluation of viral titers via plaque assays. The viral titers are expressed as mean ± s.e.m of five mice per group. Statistical significance among different vaccination groups is reported as * (*p* < 0.05), ** (*p* < 0.01), *** (*p* < 0.001), and **** (*p* < 0.0001), respectively. The experiments were repeated once with similar results.

To evaluate the protective efficacy of the glycosylated RBD mucosal vaccines, immunized mice were challenged with SARS-CoV-2 Omicron-XBB.1.5 or MERS-CoV 10 days after the second boost (an optimal time point for evaluating the protective efficacy of CoV vaccines in our pilot studies), followed by measurement of viral titers in the lungs. Among the challenged groups, the viral titers in the lungs of mice immunized with the SARS2-Omi-RBD were significantly lower after challenge with Omicron-XBB.1.5 than the viral titers in the lungs of mice receiving the MERS-RBD or the adjuvant control (Figure 5a). In addition, mice immunized with the MERS-RBD had significantly lower titers of MERS-CoV than mice immunized with the SARS2-Omi-RBD or those receiving the adjuvant control (Figure 5b). By contrast, there were no significant viral titers in the lungs of mice immunized with SARS2-WT-RBD, as compared to those of SARS2-Omi-RBD or the other groups (Figure 5b). Notably, mice receiving the cocktail mucosal vaccine had significantly lower titers of Omicron-XBB.1.5 than MERS-RBD-immunized mice or significantly lower titers than SARS2-Omi-RBD-immunized mice challenged with MERS-CoV (Figure 5a,b). These data demonstrate that while the individual glycosylated RBD proteins only protected mice against challenge with either SARS-CoV-2 (Omicron-XBB.1.5) or MERS-CoV, the cocktail protected mice against challenge with both SARS-CoV-2 and MERS-CoV, suggesting broad-spectrum protective efficacy against multiple pathogenic CoVs.

## 4. Discussion

SARS-CoV-2 has had a marked negative impact on the global economy and continues to be a threat to public health. This pathogenic CoV mutates rapidly to generate unpredictable variants. Another CoV, MERS-CoV, continues to infect humans and has a high mortality rate. Both of these CoVs cause a severe respiratory syndrome that can have marked adverse effects in many cases; therefore, there is a continuing need to develop effective mucosal vaccines that prevent virus replication at mucosal sites. Particularly, there is a need for vaccines capable of blocking infection and limiting transmission of both CoVs simultaneously [37].

Although several mucosal vaccines against SARS-CoV-2 have been developed, most are based on viral vectors, such as adenoviruses, Newcastle disease virus (NDV), murine pneumonia virus, measles virus, and vesicular stomatitis virus, or on live-attenuated viruses [38,39,40,41,42]. For example, a mucosal boosting of an adenovirus-vector-based bivalent vaccine encoding S protein of SARS-CoV-2 original strain WA.1 and Omicron-BA.5 generates airway IgG and IgA antibody responses, thereby minimizing SARS-CoV-2 Omicron-XBB.1.16 replication in the lungs and nose of non-human primates [41]. Intranasal immunization with a SARS-CoV-2 Beta-S-encoding NDV-vectored vaccine protected K18-hACE2 transgenic mice against infection by the SARS-CoV-2 Beta or Delta variants [42]. A live-attenuated SARS-CoV-2 vaccine based on codon deoptimization generated mucosal and systemic neutralizing antibodies, as well as T-cell responses, and protected intranasally immunized K18-hACE2 transgenic mice and/or hamsters against infection of the original SARS-CoV-2 strain and the Beta, Delta, and Omicron variants [43]. In addition, intranasal inoculation of mice with SARS-CoV-2 S protein together with carboxy-vinyl polymer (S-CVP) induced mucosal responses and prevented virus replication and inflammation after SARS-CoV-2-challenge [44]. Moreover, conjugation of an intranasal vaccine encoding the SARS-CoV-2 S and N proteins to a NanoSTING adjuvant elicited mucosal immune responses that protected immunized animals against challenge with several SARS-CoV-2 variants or SARS-CoV [45].

A number of vaccines against MERS-CoV have been developed, most of which are based on viral vectors, DNAs, proteins, virus-like particles, attenuated propagation-defective RNA replicons (i.e., MERS-CoV-ΔE), or mRNAs [46,47,48,49,50,51,52,53,54]. The majority target the S protein or its RBD fragment, although several target the non-S proteins, such as the nucleocapsid protein [46,47,48,49,53,55,56,57,58,59]. For example, a DNA vaccine encoding the S protein of MERS-CoV elicited immune responses in vaccinated non-human primates and protected them against infection by MERS-CoV [49]. MERS-CoV-ΔE showed protective efficacy in immunized hDPP4 transgenic mice challenged with MERS-CoV [52]. Clinical trials show that an S-targeting DNA vaccine and viral-vectored vaccines based on adenoviruses and Modified vaccinia Ankara elicit stronger immune responses at high doses than at low doses [47]. While most of these vaccines are delivered via the parenteral intramuscular immunization routes, other approaches, such as intradermal delivery of a MERS-CoV DNA vaccine via vacuum electroporation, have been attempted to generate specific humoral and cellular immune responses [49,60]. Nevertheless, mucosal vaccines that induce potent and durable mucosal responses against MERS-CoV are still under development.

Our previous studies showed that when immunization was performed using the parenteral intramuscular routes, the glycosylated original SARS-CoV-2 RBD protein (MU-RBD) induced significantly stronger neutralizing antibody responses and improved protection against SARS-CoV-2 variants and that the glycosylated MERS-CoV RBD (T579N) protein elicited significantly stronger neutralizing antibody responses and protection against MERS-CoV [35,36]. In addition, a glycosylated protein encoding the RBD of the SARS-CoV-2 Delta variant elicited mucosal immune responses and broadly neutralizing antibodies when administered intranasally that protected mice against infection by the SARS-CoV Delta variant [61]. In the present study, we designed a unique RBD-targeting cocktail comprising the three mucosal subunit vaccines consisting of the glycosylated RBDs of the original SARS-CoV-2 strain and the Omicron (XBB.1.5) variant, as well as the glycosylated RBD of MERS-CoV. Mucosal immunization of this cocktail vaccine, together with a mucosal adjuvant Poly(I:C), elicited durable mucosal and systemic antibody responses and broadly neutralizing activity against SARS-CoV-2 (the Omicron variant and the original strain) and MERS-CoV and also protected vaccinated mice against challenge with SARS-CoV-2 (Omicron-XBB.1.5 variant) and MERS-CoV. By contrast, intranasal immunization with each individual RBD protein vaccine induced immune responses and protection against either SARS-CoV-2 or MERS-CoV but not both. In addition to inducing enhanced systemic immunity via parenteral immunization, as reported before, this study confirms the ability of glycosylated proteins targeting CoV RBDs in eliciting durable, broadly effective mucosal immunity and protection against CoV infection via the mucosal route. Overall, the present report demonstrates the potency of glycosylated RBD subunit vaccines, particularly when used in combination, to provide robust mucosal immunity and protective efficacy against all of the pathogenic CoVs tested. While the vaccine-induced durable immune responses, including mucosal immunity, were measured for 40 weeks post-immunization, the protective efficacy of these mucosal vaccines were investigated at 10 days post-2nd-boost vaccination, an optimal time point for evaluating the efficacy of CoV vaccines in our previous studies. Future studies will be performed to evaluate the durable protective efficacy of these mucosal vaccines and correlate the induced immune responses with protective efficacy against CoV infection.

Simultaneous immunization with multiple immunogens (cocktail vaccination) might be an effective way to generate focused and protective immunity against multivalent viral pathogens. However, the effect of cocktail vaccination on the immune response to each individual antigen within the cocktail is hard to predict. This study has shown induction of effective and durable neutralizing antibody responses and broadly protective efficacy through a three-antigen cocktail. Other studies have found that the initial elicitation of more robust antibody responses induced by a cocktail vaccine resulted in decreased development of antibody titers over time as compared to the single antigen vaccines [62]. Therefore, more efforts will be needed to design effective combinational immunization regimens, identify the strategies of cocktail vaccines, and reveal the underlying mechanism for the development of antigen-specific immune responses.

Humoral, cellular, and mucosal immunity is important to prevent infection by SARS-CoV-2 and other CoVs. It has been reported that acute COVID-19 rapidly induced SARS-CoV-2-specific T cell responses (CD4^+^ and CD8^+^) to limit viral replication in the upper respiratory tract; these responses are independent of neutralizing antibodies [63]. CD4^+^ and CD8^+^ T cells, along with B cells and plasma cells, also protect from MERS-CoV infection in both the upper and lower respiratory tracts [64]. Glycan-engineered proteins may trigger T cell-independent activation of B cells, leading to elicitation of increased neutralizing antibodies and enhanced protective efficacy against pathogens [65,66,67]. Nevertheless, other glycan-engineered vaccines (i.e., subunit, mRNA, etc.) have shown activation of stronger cellular immune responses, as well as the induction of high-affinity antibodies and durable protection, potentially by triggering T cell-dependent B cell responses [67,68]. These studies are critical to identify the impact of glycan engineering in protein functionality and related immune responses, particularly T cell responses, a procedure that needs to be addressed in the next step. Conventional ELISA methods may not differentiate the total IgA and secretory IgA (sIgA) antibodies associated with mucosal immunity. Considering that the level of sIgA in BAL samples generally correlates to the level of total IgA, only total IgA in BAL samples was tested using the conventional ELISA in this study. Recent studies have used other assays, such as dimeric IgA (dIgA)-ELISA and dIgA-multiplex bead assay, to measure sIgA antibodies [69], which will be helpful for testing antigen-specific sIgA in future studies.

Using Poly(I:C) as a mucosal adjuvant may have some potential side effects, including potentially enhancing susceptibility to secondary pulmonary infections in the case of gram-positive bacteria or activating toll-like receptor 3 to induce inflammation and impair lung function in mice [70,71]. Here, we found that Poly(I:C) potentiates a skewed immune response towards a Th2-type (higher IgG1/IgG2a ratio) in mice (when mucosally administered with the subunit vaccines tested) without showing obvious adverse effects. When injected via an intradermal route, the Poly(I:C) adjuvant, in combination with an Alum adjuvant, is shown to potentiate a trimeric SARS-CoV-2 S protein to generate high-level IgG and neutralizing antibodies, as well as T cells toward a Th1-type profile in mice [72]. When injected via a subcutaneous route, Poly(I:C), together with IL-15, promotes the S1 protein of SARS-CoV-2 to induce high-titer and durable antibodies that bind to the RBD, S1 subunit, and full-length ectodomain of the SARS-CoV-2 protein in both C57BL/6 mice and K18-hACE2 mice [73]. Notably, no side effects are observed among injected animals. Although it has not been approved for clinical use, Poly(I:C) adjuvant, as well as its modified form (Poly-ICLC), has been widely used as an effective adjuvant in clinical trials, including cancer therapies that are safe and tolerated [74,75,76].

Mucosal immunity serves as the first line of defense against pathogens that initiate infection at mucosal surfaces, resulting in elicitation of a protective mucosal immune response [77]. However, the development of efficacious mucosal CoV vaccines for human use is challenging, due partially to the special features of mucosal tissues, which are protected by mechanical, physicochemical, and immunological barriers. To design effective mucosal vaccines, multiple factors should be taken into consideration, such as the biology features of the targeting mucosal tissues, stability of the subunit vaccines in site-specific pH and enzymatic environments, mucus interference, and delivery of vaccines to specific immune inductive sites [78]. High effective subunit vaccines, coupled with easy-to-use nasal/oral spray techniques, can be very promising and applicable for humans [79]. Encouraging preclinical findings from this and other studies developing mucosal vaccines against SARS-CoV-2 and other CoVs will provide valuable data for future clinical trials [61,80,81].

## 5. Conclusions

To summarize, we describe a distinctive approach to the design of mucosal vaccines that induce efficacious and durable mucosal immune responses and broadly neutralizing antibodies. These vaccines confer broad-spectrum protection against two important respiratory pathogenic CoVs: SARS-CoV-2 and MERS-CoV. The present approach to designing mucosal vaccines can be used to target current and future SARS-CoV-2 variants, MERS-CoV, and other mucosal viral pathogens with pandemic potential.

## Data Availability

The data related to this study are presented in the paper. Related materials will be made available under a material transfer agreement. No code was used in this study.
